# Characterization and Comparison of Human and Ovine Mesenchymal Stromal Cells from Three Corresponding Sources

**DOI:** 10.3390/ijms21072310

**Published:** 2020-03-27

**Authors:** El-Mustapha Haddouti, Thomas M. Randau, Cäcilia Hilgers, Werner Masson, Klaus J. Walgenbach, Robert Pflugmacher, Christof Burger, Sascha Gravius, Frank A. Schildberg

**Affiliations:** 1 Clinic for Orthopedics and Trauma Surgery, University Hospital Bonn, 53127 Bonn, Germany; 2 Division of Plastic, Reconstructive and Aesthetic Surgery, Department of Surgery, University Hospital Bonn, 53127 Bonn, Germany; 3 Department of Orthopaedics and Trauma Surgery, University Medical Center Mannheim of University Heidelberg, 68167 Mannheim, Germany

**Keywords:** mesenchymal stromal cells, ovine animal model, orthopedics, regenerative medicine, immunomodulation, differentiation, proliferation rate, surface markers

## Abstract

Currently, there is an increasing focus on mesenchymal stromal cells (MSC) as therapeutic option in bone pathologies as well as in general regenerative medicine. Although human MSCs have been extensively characterized and standardized, ovine MSCs are poorly understood. This limitation hampers clinical progress, as sheep are an excellent large animal model for orthopedic studies. Our report describes a direct comparison of human and ovine MSCs from three corresponding sources under the same conditions. All MSCs presented solid growth behavior and potent immunomodulatory capacities. Additionally, we were able to identify common positive (CD29, CD44, CD73, CD90, CD105, CD166) and negative (CD14, CD34, CD45, HLA-DR) surface markers. Although both human and ovine MSCs showed strong osteogenic potential, direct comparison revealed a slower mineralization process in ovine MSCs. Regarding gene expression level, both human and ovine MSCs presented a comparable up-regulation of Runx2 and a trend toward down-regulation of Col1A during osteogenic differentiation. In summary, this side by side comparison defined phenotypic similarities and differences of human and ovine MSCs from three different sources, thereby contributing to a better characterization and standardization of ovine MSCs. The key findings shown in this report demonstrate the utility of ovine MSCs in preclinical studies for MSC-based therapies.

## 1. Introduction

The bone is under constant turnover and remodeling, which is a well-regulated biological process during development and fracture healing [1,2]. However, large bone defects caused by tumor, trauma, failed arthroplasty, or osteosynthesis represent an especially challenging clinical problem. The reason for this is that bone tissues cannot afford the regeneration of large bone defects and require bone graft or biomaterials to bridge the tissue gap, restore the structural support, and sustain the physiological and regenerative process. The gold standard in reconstructing large bone defects has historically been the autologous bone graft, but it is recognized that surgical stress and bone quality of the harvested tissue are significantly limiting factors of the procedure [3]. The efficiency of hard tissue regeneration depends on a balance of osteogenic cell groups, osteoinductive stimulants, and osteoconductive matrix [4]. These biological resources, however, appear to be limited in bone grafts and in the surrounding diseased tissue. Therefore, mesenchymal stromal cell (MSC) therapies have become an area of interest as they provide a possible adjuvant for tissue regeneration. Due to their osteogenic and chondrogenic potential, they are a promising cell population which offers new ways to regenerate bone [5].

Recent studies have revealed that there are extensive interactions between bone and immune cells. New information reveals that MSCs not only contribute to tissue repair but also possess immense immunomodulatory capacity [6,7]. This immunomodulatory relevance means that MSCs are important for therapeutic modulation of disease development and degenerative processes [8,9]. Several interesting new demonstrations of the immunomodulatory capacity of MSCs showed that MSCs from different sources can influence responses and progression of various inflammatory diseases, and they have the capacity to home and integrate into impaired tissues. These immunomodulatory effects appear to be precisely coordinated with the inflammatory microenvironment [10,11].

Human (h)MSCs have been isolated from multiple different tissues [12], being well characterized and standardized according to a position statement of the International Society for Cellular Therapy (ISCT) [13,14]. However, sheep is the primary experimental large animal model for orthopedic preclinical research on bone healing, material biocompatibility, and newly developed implants. Advantages of using sheep as a primary model are their comparability to humans for multiple characteristics including body weight, bone formation, and anatomy [15,16,17,18]. Sheep are also ethically accepted and are easy to keep and handle [19]. Therefore, the characterization of MSCs from sheep is mandatory to investigate the efficacy of cell therapies for bone regeneration and implant osseointegration before clinical use of human MSCs. However, despite the convenience of utilizing sheep as a large animal model for orthopedics and the recent advantages in using MSCs, the number of studies involving ovine (o)MSCs is still very low [20].

oMSCs are poorly studied and not well characterized in comparison to hMSCs regarding their isolation, expansion, media formulation, cell surface expression, and differentiation. Due to the great opportunity and promising potential of MSCs in orthopedics, the oMSCs need standardization and direct comparison to hMSCs. Some similarities between hMSCs and oMSCs have been reported in the literature [15,20], but the reported oMSC characteristics do not meet the minimal criteria set by the ISCT for hMSCs [14]. To provide optimum use of both hMSCs and oMSCs, further efforts must be made to improve the culture conditions of MSCs, identify common surface marker expression, optimize differentiation protocols, and identify gene expression markers for lineage-specific differentiation [21]. Only through advances of both hMSCs and oMSCs can the translation of preclinical findings into clinical application come to fruition.

The current study aimed to compare hMSCs directly with oMSCs from three sources, under the same conditions, and to delineate their characteristics comparatively as set by ISCT.

## 2. Results

### 2.1. hMSC and oMSC Morphology and Proliferation Rates

**Figure 1:**
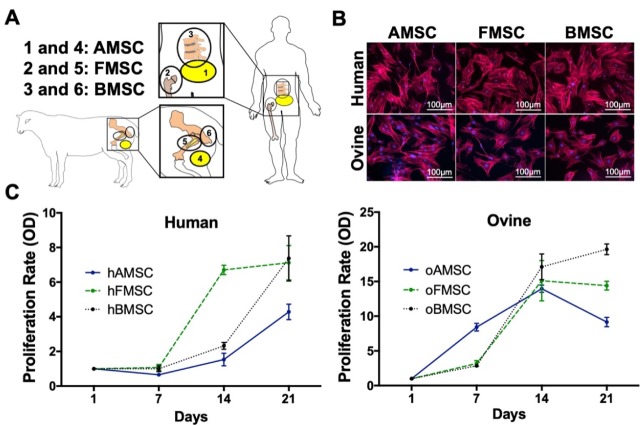
Graphical representation of the different human and ovine mesenchymal stromal cell (MSC) sources as well as their morphology and growth rate of corresponding MSCs. (**A**) MSC isolation from three corresponding human and ovine sources. Human (h)MSCs were harvested from donors undergoing abdomen liposuction cosmetic surgery (1), after hip replacement (2), and during kyphoplasty procedure (3). Ovine (o)MSCs were harvested from thigh adipose tissue (4), femoral marrow fat (5), and the tuber ischiadicum (6). (**B**) MSCs from human and ovine sources showed fibroblast-like morphology. Cytoskeleton-actin (red) and nucleus (blue). Representative pictures are shown. (**C**) MSC growth behavior was defined by measuring the optical density (OD) at the indicated time intervals. MSCs from human sources (left), MSCs from ovine sources (right). Data are expressed as average ± SEM of 3–5 donors per source. AMSC: adipose tissue-derived MSC, FMSC: femoral-derived MSC, BMSC: bone marrow-derived MSC.

Three corresponding human and ovine sources (Figure 1A) were selected for isolation of MSCs. hMSCs were harvested from donors undergoing liposuction in the abdomen (hAMSCs, adipose tissue-derived MSCs), after hip replacement (hFMSCs, femoral-derived MSCs), and during kyphoplasty procedures (hBMSCs, bone marrow-derived MSCs). oMSCs were harvested from adipose tissue (oAMSCs) in the thigh, femoral marrow fat (oFMSCs), and the tuber ischiadicum (oBMSCs). They were isolated on the basis of their ability to selectively adhere to a plastic surface. On the third day after the first culturing, the non-adherent cells were aspirated and disposed. The adherent MSCs exhibited typical mesenchymal morphology and showed flat polygonal fibroblast-like shape (Figure 1B). All MSCs from human sources (Figure 1C, left) and ovine sources (Figure 1C, right) showed a solid growth behavior. When comparing MSCs from the three human sources with each other, hFMSCs demonstrated a trend for increased proliferation in comparison to hAMSCs and hBMSCs. Comparing MSCs from the ovine sources with each other resulted only in minor differences.

### 2.2. Determination of Surface Markers

According to criteria set by the ISCT, MSCs from three human sources and their corresponding ovine sources were analyzed for surface marker expression using flow cytometry [13]. MSCs were considered to be positive for a “cluster of differentiation” (CD) surface marker if ≥95% of the MSCs expressed the marker. A negative call was given if ≤2% MSCs expressed the surface marker.

**Figure 2:**
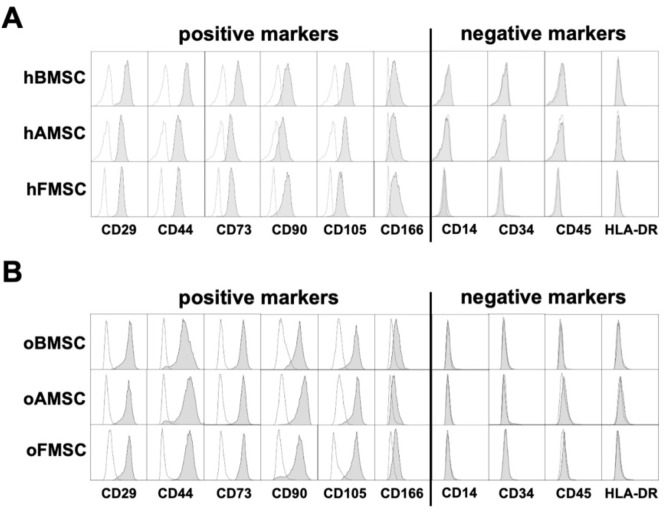
MSCs from human and ovine sources displayed common positive and negative surface markers. Surface marker expression analysis of (**A**) human and (**B**) ovine MSCs from the three corresponding sources was performed using flow cytometry. Representative histograms of 3-8 donors per source. AMSC: adipose tissue-derived mesenchymal stromal cells, FMSC: femoral-derived mesenchymal stromal cells, BMSC: bone marrow-derived mesenchymal stromal cells.

MSCs from the three human sources were positive for the following surface markers: CD29, CD44, CD73, CD90, CD105, and CD166. MSCs from the human sources were negative or low for the following surface markers: CD14, CD34, CD45, and HLA-DR (Figure 2A). Using identical surface markers for characterizing oMSCs from the three sources revealed positive staining for CD29, CD44, CD73, CD90, CD105, and CD166, and negative staining for CD14, CD34, CD45, and HLA-DR (Figure 2B). Therefore, all sources of human and ovine MSCs showed the same surface marker pattern and fulfilled the major positive and negative markers defined by ISCT.

### 2.3. Immunomodulatory Capacity

To compare the ability of MSCs from human and ovine sources to exhibit immune inhibitory properties, MSCs from the three human sources and MSCs from the three corresponding ovine sources were tested. To evaluate this capacity, MSCs were measured on their inhibitory effect on lymphocyte proliferation. Carboxyfluorescein succinimidyl ester (CFSE)-labelled human and ovine lymphocytes were stimulated with phorbol myristate acetate (PMA)/ionomycin in the absence or presence of hMSCs or oMSCs, respectively. The lymphocyte proliferation was analyzed by flow cytometer using CFSE dilution after 3 days.

**Figure 3:**
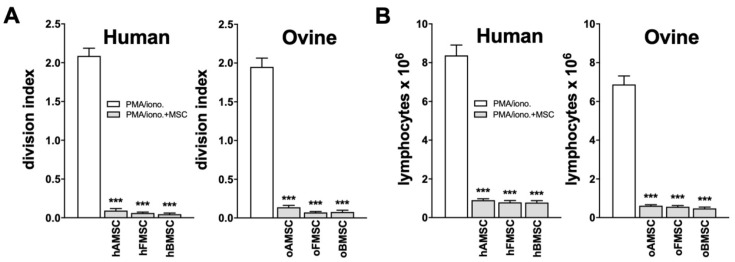
Immunomodulatory capacity of MSCs from human and ovine sources. Human and ovine MSCs from the three corresponding sources showed comparable immunomodulatory capacity by suppressing the proliferation of human and ovine lymphocytes, respectively. Carboxyfluorescein succinimidyl ester (CFSE)-labelled human and ovine lymphocytes were stimulated with PMA/ionomycin in the absence or presence of MSCs. (**A**) Calculations of division index and (**B**) total cell numbers of human and ovine lymphocytes are shown. Data are expressed as average ± SEM of 3-6 donors per source. *** *p* < 0.001, Mann–Whitney U test.

Proliferation of both human and ovine lymphocytes was clearly suppressed by MSCs from both human and ovine sources, respectively (Figure 3A). In detail, although all lymphocytes proliferated in the presence of PMA/ionomycin, the presence of MSCs completely inhibited lymphocyte proliferation and reduced the division index to background levels (Figure 3A). Additionally, and for verification, total lymphocyte number was determined. Absolute lymphocyte numbers confirmed MSC immunosuppression capacity, clearly indicated by inhibition of lymphocyte proliferation, as shown by cell counts in the presence of MSCs (Figure 3B).

### 2.4. Differentiation towards Adipogenic and Chondrogenic Lineages

**Figure 4:**
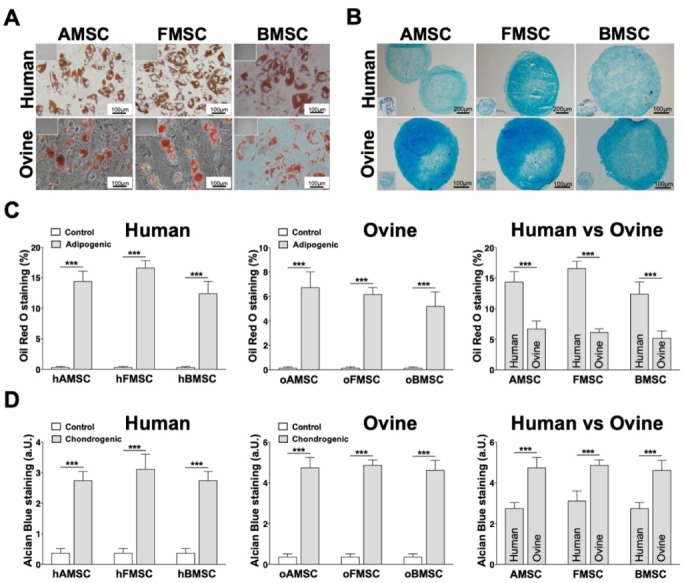
Adipogenic and chondrogenic differentiation of MSCs from human and ovine sources. MSCs from three human and ovine corresponding sources were induced towards the (**A**) adipogenic and (**B**) the chondrogenic lineages for 21 days. Culturing medium without any supplements was used as control. (**A**) The adipogenic differentiation was confirmed via Oil Red O and (**B**) the chondrogenic differentiation via Alcian Blue stainings. Controls are indicated in the corners. (**C**) The adipogenic differentiation rate of MSCs from human sources (left), ovine sources (middle), and human versus ovine sources (right) was evaluated by measuring the percentage of cells stained positive using the cellSens Dimension software. (**D**) The chondrogenic differentiation rate of MSCs from human sources (left), ovine sources (middle), and human versus ovine sources (right) was depicted by setting a semi-quantitative score based on the intensity of Alcian Blue staining: (1) very weakly positive, (2) weakly positive, (3) moderately positive, (4) markedly positive, and (5) strongly positive. Data are either representative pictures or expressed as average ± SEM of 3–4 donors per source. *** *p* < 0.001, Student’s two-tailed unpaired *t*-test.

MSCs from the three human and ovine sources were investigated for their differentiation potential towards the adipogenic and chondrogenic lineages. In addition to their morphological changes, visible lipid-rich vacuoles accumulated in MSCs from all sources during adipogenic differentiation. Confirmation of adipogenic differentiation was completed via Oil Red O staining at the end of induction time (Figure 4A). For the chondrogenic differentiation, both hMSCs and oMSCs from all sources showed typical characteristics of glycosaminoglycan matrix when stained with Alcian Blue, 3 weeks after induction (Figure 4B). All controls were cultured under the same conditions, without supplementation, and did not result in adipogenic nor chondrogenic differentiation (Figure 4A,B, inserts).

To further analyze the adipogenic differentiation potential, the Oil Red O staining intensity of MSCs from human and ovine sources was evaluated by quantifying the amount of positively stained cells per image. This unbiased quantification approach confirmed a very solid adipogenic differentiation of all induced MSCs in comparison to the controls (Figure 4C left and middle). Interestingly, hMSCs from all three sources showed a significantly higher adipogenic differentiation rate compared to oMSCs from all three corresponding sources (Figure 4C, right). Further, we also quantified the chondrogenic differentiation rate of MSCs from human sources and MSCs from ovine sources by a semi-quantitative score based on Alcian Blue staining. The quantification of Alcian Blue staining indicated a clearly significant chondrogenic rate for both induced hMSCs (Figure 4D, left) and oMSCs (Figure 4D, middle) compared to their corresponding controls. MSCs derived from ovine sources showed a significantly higher chondrogenic differentiation rate compared to MSCs from human sources (Figure 4D, right).

### 2.5. Assessment of Osteogenic Differentiation

**Figure 5:**
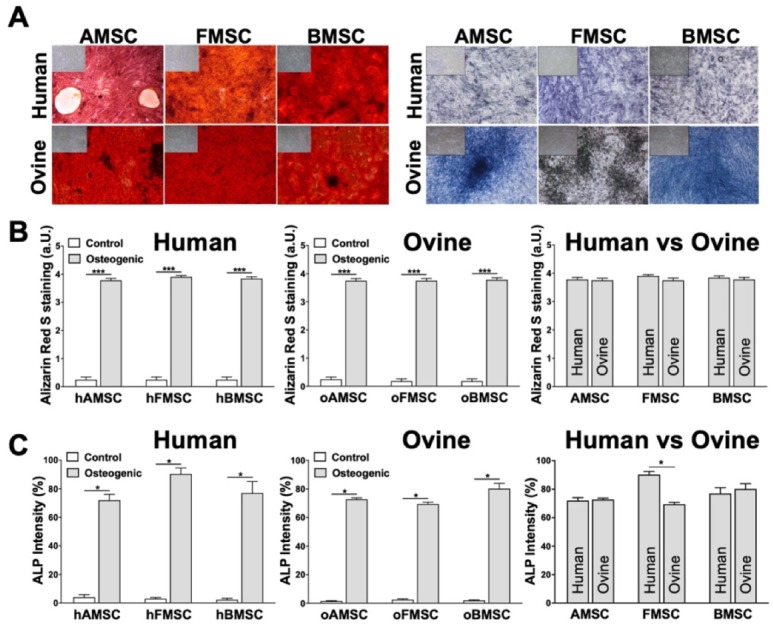
Strong mineralization rate and significant relative alkaline phosphatase intensity of MSCs from human and ovine sources differentiated towards the osteogenic lineage. hMSCs and oMSCs were induced towards the osteogenic lineage for 21 days. Culturing medium without any supplements was used as control. (**A**) The mineralization was confirmed via Alizarin Red S (left) and alkaline phosphatase (ALP) (right) staining. Controls are indicated in the top left corners. (**B**) The mineralization rate of MSCs from human sources (left), ovine sources (middle), and human versus ovine sources (right) was depicted by setting a semi-quantitative score based on the intensity of Alizarin Red S staining: (0) negative, (1) weakly positive, (2) moderately positive, (3) markedly positive, or (4) strongly positive. **(C)** The relative ALP staining intensity of MSCs from human sources (left), ovine sources (middle), and human versus ovine sources (right) was evaluated by measuring the percentage of cells stained positive using the cellSens Dimension software. White bars indicate control, grey bars indicate osteogenic induction. Data are either representative pictures or expressed as average ± SEM of 3–6 donors per source. * *p* < 0.05, *** *p* < 0.001, Mann–Whitney U test.

For the osteogenic lineage, all MSCs were induced for 21 days and the osteogenic differentiation was confirmed via Alizarin Red S (Figure 5A, left) and alkaline phosphatase (ALP) staining (Figure 5A, right). For control cultures, identical conditions were utilized, without supplementation, and stained negative for both Alizarin Red S and ALP (Figure 5A, inserts in the top left corners).

The mineralization rate of analyzed MSCs from human sources (Figure 5B, left) and MSCs from ovine sources (Figure 5B, middle) was quantified by a semi-quantitative score based on Alizarin Red S staining, which quantifies the mineralized matrix secreted by differentiating MSCs towards osteoblasts. This staining resulted in clearly higher values for both toward osteogenic differentiation-induced hMSCs and oMSCs compared to the corresponding controls (Figure 5B, left and middle). Comparable human and ovine sources were found to have similar mineralization rates of AMSCs, FMSCs, and BMSCs (Figure 5B, right).

The relative ALP staining intensity of MSCs from human sources (Figure 5C, left) and MSCs from ovine sources (Figure 5C, middle) was also evaluated. ALP is an early expressed osteogenic protein marker that accumulates in the membrane and can be used to confirm osteogenic differentiation. By scoring the percentage of cells positive for ALP, we determined that the relative ALP staining intensity was comparable between all MSCs from human and ovine sources, except the human FMSC source that showed significantly increased staining (Figure 5C, right).

**Figure 6:**
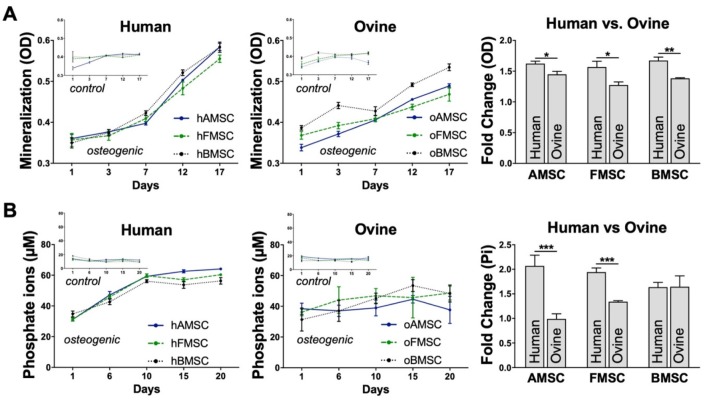
Mineral deposition and phosphate ion release by MSCs from human and ovine sources. MSCs from human and ovine sources were induced towards the osteogenic lineage. Culturing medium without any supplements was used as control. (**A**) The mineral deposition of MSCs from human sources (left) and MSCs from ovine sources (middle) was assessed by optical density (OD) measurement at different time intervals, as indicated. The overall mineralization fold change of MSCs from human and ovine sources was mediated using the ratio d17/d1 (right). (**B**) The osteogenic differentiation process of MSCs from human sources (left) and MSCs from ovine sources (middle) was assessed by measuring the inorganic free phosphate ions (Pi) released into the supernatant at different time intervals. The overall phosphate ion release fold change of MSCs from human sources and MSCs from ovine sources was mediated using the ratio d20/d1 (right). Data are expressed as average ± SEM of 3-6 donors per source. * *p* < 0.05, ** *p* < 0.01, *** *p* < 0.001, Student’s two-tailed unpaired *t*-test.

Moreover, the mineralization process was further assessed by optical density (OD) of monolayer cultures using a microplate reader at different time intervals during the induction period. Mineralized areas of monolayer cell cultures appear darker when measuring the OD [22], which makes this a fast approach to investigate the osteogenic differentiation. With the help of this assay, we could show that mineralized areas in osteogenic lineage-induced cells had an increased OD compared to control cultures in both hMSCs and oMSCs. A significant shift at day 7 was seen in the osteogenic lineage-induced MSCs from both human and ovine sources. This shift continued to increase steadily compared to the corresponding controls (Figure 6A, left and middle). The overall fold change was mediated by using the ratio d17/d1 and demonstrated that the mineralization process was slightly increased in all three hMSC sources compared to the corresponding oMSC sources (Figure 6A, right), indicating a slower mineralization process for oMSCs. There were clear calcium deposits from both human and ovine sources; their mineralization was confirmed via Alizarin Red S staining, demonstrating successful osteogenic differentiation. Control MSCs showed no calcium deposits from either human or ovine sources and stained negative for Alizarin Red S.

In addition to assessing the mineralization process, the osteogenic differentiation of MSCs from both human and ovine sources was monitored through measurement of inorganic free phosphate ions (Pi) released into the supernatant at different time intervals during the induction period. A distinct increase in Pi at all time points was noted in all osteogenic lineage-induced MSCs compared to their corresponding controls (Figure 6B, left and middle). The Pi fold change of MSCs from human sources and MSCs from ovine sources was mediated by using the ratio d20/d1 and showed that MSCs from two human sources (hAMSCs, hFMSCs) were approximately one-fold higher compared to the corresponding ovine sources (oAMSCs, oFMSCs). Interestingly, MSCs from the human BMSC source showed no significant difference compared to the ovine BMSC source (Figure 6B, right).

### 2.6. Osteogenic Lineage-Specific Gene Expression

Finally, MSCs from the three human and ovine sources were induced towards the osteogenic lineage for 21 days to allow for gene expression quantitation. Controls were cultured in medium without supplementation. The osteogenic differentiation was assessed using RT-PCR to investigate the relative mRNA expression of two osteogenic lineage-specific genes, Runx2 and Col1A. These were quantitated on day 1 and day 21 of the osteogenic induction.

**Figure 7:**
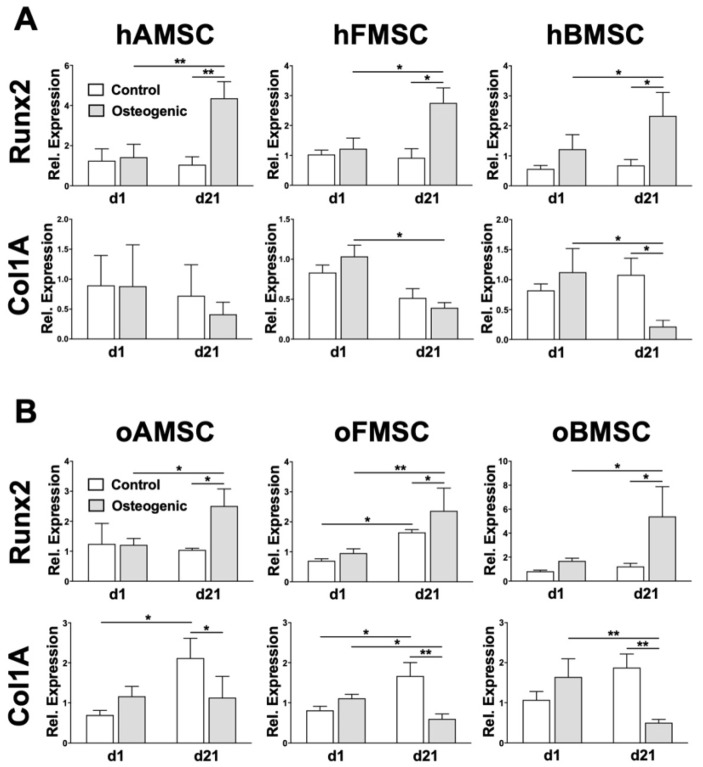
MSCs from (**A**) human and (**B**) ovine sources expressed common osteogenic gene marker Col1A and Runx2. MSCs from three corresponding human and ovine sources were induced towards the osteogenic lineage for 21 days. Culturing medium without any supplements was used as control. The relative expression of Col1A and Runx2 genes was investigated on day 1 and day 21. Data analysis was performed using delta-delta-Ct (ddCT) values normalized to glyceraldehyde-3-phosphate dehydrogenase (GAPDH) and the corresponding samples harvested on day 0. Data are expressed as average ± SEM of 3–5 donors per source. * *p* < 0.05, ** *p* < 0.01, two-way ANOVA.

Initially, the mRNA expression of Runx2 was slightly up-regulated on day 1 after induction in FMSCs and BMSCs from both human and ovine sources compared to controls (Figure 7A,B, top panels). However, on day 21 of induction, mRNA expression of Runx2 was up-regulated in all MSCs from both human and ovine sources compared to the corresponding controls as well as compared to day 1 of induction (Figure 7A,B, top panels). The second osteogenic lineage-specific gene, Col1A, showed no significant change on day 1, but was clearly down-regulated on day 21 in both MSCs from human and ovine sources compared to the corresponding controls (Figure 7A,B, bottom panels), suggesting a feedback down-regulation as has been described previously both at mRNA [23] and protein level [24].

## 3. Discussion

MSCs play a key role in processes important for health and disease [25]. Considering their role in multiple tissues and organs, advanced studies are dedicated to deciphering the basic biology and potential clinical applications of MSCs [26,27,28,29,30]. Some advantages to MSC manipulation are ease of harvest, minimal ethical concern, and that they do not tend to form tumors. They also exhibit the unique property of self-renewal and the remarkable ability to differentiate into diverse cell types including adipocytes, osteoblasts, and chondrocytes when cultured under specific growth conditions in vitro [31]. Additionally, MSCs from different sources have been demonstrated to possess a significant immunomodulatory capacity. In inflammatory diseases, MSCs uniquely respond by homing and integrating into impaired tissues [10,11,27]. These unique immunomodulatory properties establish MSCs as a cell type of primary interest for clinical advancement in many fields of research [32]. More specifically, MSCs show great potential as future therapeutic option in the pathophysiology of orthopedic injury and disease, and MSCs have been identified for their promising potential in regenerative medicine [33].

Human MSCs have been well characterized and standardized and their minimal criteria fulfillment have been outlined in a position statement of the ISCT in 2006 [13,14]. oMSCs, however, are poorly characterized, and remain un-standardized [34,35,36,37]. Recently, several interesting studies have partially characterized bone marrow-derived MSCs for bone formation in a sheep model [38]. The reported results from that study demonstrated that oMSCs have a high impact on implants and bone-engineered tissue testing in sheep; therefore, oMSCs have been further investigated regarding their growth and differentiation potential with various culture media and differentiation protocols. Interestingly, it has been reported that proliferation, surface marker expression, and differentiation of oMSCs are culture medium-dependent [39], which further underlines the need of a continued thorough characterization and comparison of human and ovine MSCs.

The first study comparing human and ovine bone marrow- and adipose tissue-derived MSCs by Kalaszczynska et al. investigated the MSC responses to various osteogenic differentiation media. The mineralization of oMSCs was not possible though, which was in stark contrast to hMSCs [40]. Later studies attempted osteogenic differentiation of human and ovine bone marrow-derived MSCs by utilizing different protocols including supplementation with bone morphogenetic protein 2 (BMP-2). Again, oMSCs responded poorly compared to hMSCs [21]. Most recently, a thorough review of oMSC isolation and characterization was published, which discussed the previously conflicting results and challenges in oMSCs. The review also delineated the important similarities between hMSCs and oMSCs [41].

In the current study, MSCs were isolated from three human and three corresponding ovine sources and expanded by applying the same protocol. All MSCs exhibited typical fibroblast morphology with spindle shape and showed robust proliferation behavior. Further confirming previous reports pertaining to MSC proliferation by other investigators [21,42], research found that proliferation of MSCs from ovine sources was 2-3-fold higher when compared to MSCs from the corresponding human sources cultured under same conditions. However, a direct comparison of the growth behavior between human and ovine MSCs is only possible with limitations, as there are still several open questions, such as whether isolated MSCs have the same developmental stage or how age affects this interspecies comparison.

In contrast to hMSCs, the cell surface expression profile of oMSCs has not previously been well characterized. A recent literature review [41] of MSC comparisons indicated that the field is still missing consensus for a common surface marker panel. Although some studies found relevant expression of CD44, CD73, CD90, CD105, CD166, and CD271 [43,44], other studies have reported the expression of CD29 and absence of CD90 in oMSCs [35,39], thereby yielding a sum of conflicting reports. In our study, CD29, CD44, CD73, CD90, CD105, and CD166 were identified as positive markers in both hMSCs and oMSCs, in addition to CD14, CD34, CD45, and HLA-DR as common negative surface markers for both hMSCs and oMSCs. These results prove oMSCs to be even more comparable to hMSCs and contribute to a long discussion about their MSC-specific surface markers.

Immunomodulation is important for therapeutic advances, yet most of the reported immunomodulatory properties of MSCs have been investigated using human and mouse MSCs [45]. To date, only a limited number of studies has demonstrated the immunosuppressive potential of oMSCs [46]. Our direct comparison now demonstrates that MSCs from both human and ovine sources show comparable immunomodulatory capacity by suppressing lymphocyte proliferation.

In another confirmation of current literature [39,42,47], our study describes the adipogenic differentiation potential of MSCs from all three human and ovine sources as seen by the accumulation of large lipid-rich vacuoles. Interestingly, in our direct comparison, hMSCs showed significantly increased adipogenic potential in comparison to oMSCs. Previous studies have already shown that oBMSCs show no or only low adipogenic differentiation potential, even if different protocols were used [39,42]. However, to our knowledge, a direct comparison of adipogenic differentiation of human and ovine MSCs from several sources has not been reported before. In contrast to adipogenic differentiation, our comparative study demonstrated that oMSCs have a significantly higher capacity for chondrogenic differentiation compared to hMSCs. This evidence is of particular importance as it aids MSC-based strategies for cartilage repair, a subdiscipline that has increasingly been focused on the comparison of human and ovine MSCs. Such direct comparisons are needed for translating the findings in sheep cartilage repair models into the clinic for human use [41,47].

Further, our study demonstrated that both human and ovine MSCs from the three corresponding sources showed strong mineralization rates. It also indicated significant relative ALP intensity after differentiation towards the osteogenic lineage. Recently, conflicting results arose when human and ovine MSCs were compared for their mineralization capacity using β-glycerophosphate and sodium dihydrogen phosphate (NaH_2_PO_4_) as a source of phosphate ions [21,40]. hBMSCs have been reported to mineralize in the presence of β-glycerophosphate, but not with NaH_2_PO_4_, whereas hAMSCs behaved the opposite way. Interestingly, oBMSCs and oAMSCs were able to mineralize in the presence of NaH_2_PO_4_ but not with glycerophosphate [40]. In another study, the phosphate ion sources NaH_2_PO_4_ and glycerophosphate were combined with BMP-2, and osteogenic potential of hBMSCs and oBMSCs were investigated. Although oBMSCs responded poorly compared to hBMSCs, the study also revealed that matrix deposition was improved in NaH_2_PO_4_ and showed no mineralization in β-glycerophosphate [21]. These studies nicely foster the need for a reliable osteogenic induction for both human and ovine MSCs.

In our study, we evaluated the β-glycerophosphate-mediated osteogenic differentiation of hMSCs and oMSCs from three sources at different time points. Overall, strong mineralization rates could be seen in MSCs from both human and ovine corresponding sources using Alizarin Red S staining. This report is the first assessment of the mineralization process of MSCs from three different ovine sources in comparison to MSCs from three corresponding human sources and therefore lays the fundament for future studies utilizing the osteogenic capacity of oMSCs. As Alizarin Red S staining, however, is suboptimal to detect delicate differences in osteogenic differentiation, we employed further sophisticated assays to quantify the mineralization process. In detail, we utilized a methodology to analyze the osteogenic process by monitoring the OD of monolayer cultures of hMSCs and oMSCs, as described previously by Loebel et al. [22,48]. This technique can be used as an additional measure at early stages of mineralization during osteogenic differentiation and is particularly advantageous because there is no need for staining or biochemical assays in contrast to assays relying on Alizarin Red S. Although the Alizarin Red S staining did not result in significant differences, the OD assay indicated significantly higher mineralization rates in hMSCs from all three sources compared to the corresponding oMSCs. This could be explained by the sensitivity of the OD measurements and the fact that MSCs responded differently to the osteogenic induction medium, suggesting that oMSCs possess a reduced mineralization capacity.

Further, we analyzed the mineralization process by measuring the free phosphate ion release at various time points during the osteogenic differentiation, as they play a crucial role in bone matrix mineralization [49]. Both hMSCs and oMSCs from the corresponding sources demonstrated a distinct increase of phosphate ion release during the osteogenic lineage progression. Calculated fold changes indicated higher phosphate ion release in two human sources, AMSCs and FMSCs, when compared to their corresponding ovine sources. When comparing phosphate ion release of hBMSCs and oBMSCs, however, there was no significant difference.

Moreover, osteogenic differentiation was analyzed at the gene expression level utilizing RT-PCR at two different time points of the osteogenic differentiation process. Clearly, MSCs from both human and ovine sources demonstrated an increase of the osteogenic marker Runx2 at day 21 compared to day 1. Col1A demonstrated a slight increase at induction day 1 but showed a significant decrease at day 21. These relative mRNA expression differences are in line with the reported findings in hMSCs [23,50,51]; however, thus far there has not been a consensus for oMSCs [21,47].

To our knowledge, we reported for the first time an investigation characterizing and comparing hMSCs from three sources with oMSCs from three corresponding sources, side by side under the same conditions and using only one protocol. Here, we specifically assessed the mineralization process via OD measurement, free phosphate ion release, and osteogenic gene expression. Common positive and negative surface markers were also identified on hMSCs and oMSCs from the three sources. In summary, this direct comparison defines phenotypic similarities and differences of human and ovine MSCs from three different sources, thereby contributing to a better characterization and standardization of ovine MSCs. The key findings supplied in this report demonstrate the utility of ovine MSCs in preclinical studies for MSC-based therapies.

## 4. Materials and Methods

### 4.1. Tissue Donors and Study Design

Recruitment of human subjects was approved by the ethics committee of the University Hospital Bonn (project IDs: 122/09 and 102/19) and was conducted in accordance with the approved guidelines as well as the declaration of Helsinki. All animal experiments were approved by the official state animal care and use committee (LANUV NRW, 8.87-50.10.35.08.308). Experiments were performed in accordance with the German federal law regarding the protection of animals, institutional guidelines, and the criteria in “Guide for the Care and Use of Laboratory Animals” (National Institutes of Health publication 8th Edition, 2011) were followed.

This study was designed to characterize and compare human and ovine MSCs from three sources under the same conditions. Due to the anatomical structure and musculoskeletal function, we defined corresponding sources (Figure 1A) for the isolation of adipose tissue-derived MSCs (hAMSCs), femoral-derived MSCs (hFMSCs), and bone marrow-derived MSCs (hBMSCs). hMSCs were harvested from donors undergoing liposuction in the abdomen (hAMSCs, *n* = 4), after hip replacement (hFMSCs, *n* = 8), and during kyphoplasty procedures (hBMSCs *n* = 5) (Figure 1A). Ovine subjects, more specifically, Merino sheep, had oMSCs harvested from adipose tissue in the thigh (oAMSCs, *n* = 4), femoral marrow fat (oFMSCs, *n* = 4), and the tuber ischiadicum (oBMSCs, *n* = 7). After successful isolation of human and ovine MSCs from the indicated sources, we investigated their morphology, proliferation rate, surface marker expression, immunomodulatory capacity, and differentiation potential towards the three lineages (adipogenic, chondrogenic, and osteogenic). More detailed experiments were performed to elucidate and compare the osteogenic differentiation process, including measurement of the mineralization process via optical density (OD), quantification of the free phosphate ion release, and RT-PCR.

### 4.2. MSC Isolation and Culture

hAMSCs and oAMSCs (Figure 1: 1 and 4) were isolated by mixing adipose tissues with pre-warmed (37 °C) Dulbecco’s phosphate-buffered saline (DPBS; 1:1) and shaken thoroughly, followed by room temperature incubation for 30 min. The bottom fluid phase was then aspirated and DPBS was added to the upper phase (1:1). Vigorous shaking and collagenase digestion (0.15 U/mL; Sigma Aldrich, Darmstadt, Germany) followed for 60 minutes in a shaking water bath at 37 °C. Human and ovine FMSCs (Figure 1: 2 and 5) and BMSCs (Figure 1: 3 and 6) were isolated through gradient centrifugation (800× *g* for 30 min without brake) using Biocoll separating solution (Biochrom AG, Berlin, Germany). All human and ovine cells were plated in cell culture flasks (Greiner Bio-One GmbH, Frickenhausen, Germany) with Dulbecco’s modified Eagle’s medium (DMEM) (Gibco by Life Technologies, Darmstadt, Germany) containing 10% serum, 1% L-glutamine, and 1% penicillin-streptomycin (Biochrom AG, Berlin, Germany). Incubation took place under standard conditions at 37 °C in a humidified atmosphere with 5% CO_2_.

### 4.3. MSC Morphology

All cells, hMSCs and oMSCs, were cultured as a monolayer and grown to optimal confluency, fixed with 4% paraformaldehyde (PFA) (5 min), followed by a washing step with DPBS. Next, MSCs were treated with Triton X-100 for 5 min for membrane permeabilization. Actin stock solution (Abcam plc, Cambridge, United Kingdom) was diluted (1:1000) and applied to MSCs for 10 min while nuclear counterstains were completed with 4′,6-diamidino-2-phenylindole (DAPI).

### 4.4. MSC Proliferation

The proliferation and growth characteristics of human and ovine MSCs were investigated. Cells were plated in 96-well plates as a monolayer at a density of 2 × 10^3^ cells per well with standard culture medium for 21 days. Every third day of the growth period, medium was changed. At the indicated time points, cellular optical density (OD) was determined at 570 nm according to the manufacturer’s instructions utilizing the MTT cell proliferation assay (Boster Biological Technology Co., Ltd, Pleasanton, CA, USA).

### 4.5. Immune Modulation

For examination of MSC immune inhibitory capacity, hMSCs and oMSCs were seeded in 24-well plates and cultured to confluence. For the isolation of peripheral blood mononuclear cells (PBMC), human and ovine peripheral blood was mixed with DPBS (1:1), then gently layered on a Biocoll separating solution (Biochrom AG, Berlin, Germany) and centrifuged at 800× *g* for 30 min without brake. Mononuclear cells were collected from the liquid interface and washed with DPBS. Without further purification, the naive freshly isolated human and ovine lymphocytes were labelled with CFSE (Molecular Probes, Leiden, Netherlands) and added to hMSCs or oMSCs. Lymphocytes were stimulated with PMA/ionomycin (Thermo Fisher Scientific, Karlsruhe, Germany). After 3 days, flow cytometry was performed to quantify lymphocyte proliferation by CFSE dilution, as described previously [52,53], and data were analyzed using FlowJo software 10 (BD Biosciences, Heidelberg, Germany).

### 4.6. MSC Surface Marker Expression

Flow cytometry was used to evaluate surface marker expression on MSCs. MSCs were resuspended in DPBS with 1% fetal bovine serum (FBS)/2 mM ethylenediaminetetraacetic acid (EDTA) and were stained with saturating concentrations of antibodies (Miltenyi Biotec, Bergisch Gladbach, Germany) for 20 min. Flow cytometry data were acquired on a BD FACS Canto ll flow cytometer (BD Biosciences, Heidelberg, Germany) and analyzed using FlowJo software (BD Biosciences, Heidelberg, Germany). Human and ovine MSCs were tested for CD14, CD29, CD34, CD44, CD45, CD73, CD90, CD105, CD166, and HLA-DR. All antibodies have been validated to work in sheep by previous papers and/or according to manufacturers’ instructions.

### 4.7. Adipogenic Differentiation

For adipogenic lineage differentiation, hMSCs and oMSCs at a density of 1 × 10^4^ cells/cm^2^ were induced through incubation with culture medium supplemented with 1 µM dexamethasone, 1 µM insulin, and 200 µM indomethacin (Sigma Aldrich, Darmstadt, Germany) for 21 days. Culture medium lacking supplementation was used as control. At the end of the adipogenic differentiation period, cells were washed with DPBS, fixed with 4% formalin at 37 °C for 30 min, and incubated with 0.1% Oil Red O staining (Sigma Aldrich, Darmstadt, Germany) for 30 min. A collection of images was taken using light microscopy and the relative intensity of the adipogenic staining was quantified using the cellSens Dimension software (Olympus Corporation, Hamburg, Germany).

### 4.8. Chondrogenic Differentiation

The chondrogenic lineage differentiation of hMSCs and oMSCs was induced using high-glucose DMEM medium supplemented with 1 µg/mL insulin, 1 ng/mL transferrin, 1 ng/mL sodium selenite, 0.1 µM dexamethasone, 50 µM 2-phosphate-L-ascorbic acid trisodium salt, and 10 ng/mL transforming growth factor beta-1 (TGF-β1) (Sigma Aldrich, Darmstadt, Germany). MSCs were cultured on agarose gel to allow self-formation of 3D microspheres, as described previously [54]. On top of 60 µL solidified 2% agarose in 200 µL corresponding medium, 2.5 × 10^4^ cells were cultured for 21 days. The 3D microspheres were fixed with 4% PFA overnight at 4 °C and cut into 15 µm cryosections (Microm 550, Thermo Scientific, Schwerte, Germany). Staining was completed with Alcian Blue (Sigma Aldrich, Darmstadt, Germany). The chondrogenic differentiation rate was analyzed by setting a semi-quantitative score based on the intensity of Alcian Blue staining: (1) very weakly positive, (2) weakly positive, (3) moderately positive, (4) markedly positive, or (5) strongly positive.

### 4.9. Osteogenic Differentiation

Induction towards the osteogenic lineage was performed by supplementing culture medium with 0.1 µM dexamethasone, 10 mM β-glycerophosphate disodium salt hydrate, and 50 µM 2-phosphate-L-ascorbic acid trisodium salt (Sigma Aldrich, Darmstadt, Germany) for both hMSCs and oMSCs. MSCs were seeded at a density of 10^4^ cells/cm^2^ and cultured for 21 days. When differentiation was complete, cells were fixed in 4% formalin and stained with 40 mM Alizarin Red S (Sigma Aldrich, Darmstadt, Germany) and ALP (Dako, Hamburg, Germany). ALP staining was performed using the 5-bromo-4-chloro-3-indolyl phosphate (BCIP)/nitro blue tetrazolium (NBT) substrate system (Dako, Hamburg, Germany) according to the manufacturer´s instructions. A collection of images of all samples was taken by using a light microscope and the mineralization rate was depicted by setting a semi-quantitative score based on the intensity of Alizarin Red S staining: (0) negative, (1) weakly positive, (2) moderately positive, (3) markedly positive, or (4) strongly positive. The relative ALP staining intensity was analyzed by measuring the percentage of stained cells using the cellSens Dimension software (Olympus Corporation, Hamburg, Germany).

### 4.10. Optical Density and Free Phosphate Measurements

MSCs were induced towards the osteogenic lineage at a density of 10^4^ cells/cm^2^ in 96-well plates. Culture medium free of supplementation was used as control, and the medium was replaced every second or third day. The mineralization process was assessed by measuring the optical density (OD) adapted from Loebel et al. [22,48]. Briefly, the OD absorbance (450 nm) was used to evaluate the osteogenic differentiation of MSC monolayer cultures at the indicated time intervals (TECAN, Männedorf, Switzerland). Following the OD measurement, cells were washed with DPBS, and fresh medium was added to continue the differentiation process until the next measurement. The acquired OD values were corrected by the measured values of the corresponding control and osteogenic differentiation medium. Inorganic phosphate ion (Pi) release was determined in cell culture supernatant at the indicated time points by using the Malachite Green Phosphate Assay Kit (Sigma Aldrich, Darmstadt, Germany). The amounts of released free phosphate was corrected by the measured values of the corresponding control and osteogenic differentiation medium.

### 4.11. Real-Time Polymerase Chain Reaction (RT-PCR)

After osteogenic lineage induction, described above, total RNA was extracted using TRIzol Reagent (Ambion, Life Technologies, Darmstadt, Germany) at indicated time points. Briefly, cells were washed with PBS and lysed in TRIzol following chloroform/isopropanol (ratio 24:1) treatment according to the manufacturer’s instructions (PanReac AppliChem, Darmstadt, Germany).

After centrifugation, the upper phase with RNA was collected and precipitated by adding isopropanol. Washes with ethanol (80%) followed the precipitation. The Transcriptor First Strand cDNA Synthesis Kit (Roche Diagnostics GmbH, Mannheim, Germany) was utilized for complementary DNA (cDNA) synthesis. RT-PCR was performed using a LightCycler 480 II and SYBR Green I Master according to the manufacturer’s instructions (Roche Diagnostics GmbH, Mannheim, Germany). RT-PCR primer sequences are outlined in Table 1. Data analysis was performed using delta-delta-Ct (ddCT) values obtained by normalization to glyceraldehyde-3-phosphate dehydrogenase (GAPDH) and the corresponding samples harvested on day 0.

### 4.12. Statistics

Data were collected in Microsoft Excel (Microsoft Corporation, Richmond, USA), and statistical analysis was carried out using GraphPad Prism 7 (GraphPad, La Jolla, CA, USA). The Shapiro–Wilk test was used to test for normal distribution. For data with Gaussian distribution, two-tailed, unpaired Student’s *t*-test or two-way ANOVA were used. For non-Gaussian distributed data, Mann–Whitney U testing was used. Significance levels are marked as * *p* < 0.05, ** *p* < 0.01, *** *p* < 0.001.

## Figures and Tables

**Table 1:** Real-time polymerase chain reaction (RT-PCR). Primers used for the relative osteogenic gene expression of Col1A and Runx2 in hMSCs and oMSCs.

Gene	Human	Ovine
GAPDH	fwd: 5′CTCTGCTCCTCCTGTTCGAC3′ rev: 5′ACCAAATCCGTTGACTCCGA3′	fwd: 5′TCACCATCTTCCAGGAGCGA3′ rev: 5′GGTGCAGAGATGATGACCCT3′
Col1A	fwd: 5′TGCTCGTGGAAATGATGGTG3′ rev: 5′CCTCGCTTTCCTTCCTCTCC3′	fwd: 5′CATGACCGAGACGTGTGGAA3′ rev: 5′CATTCGTCCGTGGGGACTTT3′
Runx2	fwd: 5′GCGCATTCCTCATCCCAGTA3′ rev: 5′GGCTCAGGTAGGAGGGGTAA3′	fwd: 5′ CCGCCGGACTCGAACTG3′ rev: 5′GAGAGGCGCAGGTCTTGATG3′

## Abbreviations

ALP	Alkaline phosphatase
AMSC	Adipose tissue-derived mesenchymal stromal cells
BMP-2	Bone morphogenetic protein 2
BMSC	Bone marrow-derived mesenchymal stromal cells
CD	Cluster of differentiation
cDNA	Complementary deoxyribonucleic acid
CFSE	Carboxyfluorescein succinimidyl ester
Col1A	Collagen, type I, alpha 1
DAPI	4′,6-Diamidino-2-phenylindole
ddCT	Delta-delta-Ct
DMEM	Dulbecco’s modified Eagle’s medium
DPBS	Dulbecco’s phosphate-buffered saline
EDTA	Ethylenediaminetetraacetic acid
FMSC	Femoral-derived mesenchymal stromal cells
GAPDH	Glyceraldehyde-3-phosphate dehydrogenase
hAMSC	Human adipose tissue-derived mesenchymal stromal cells
hBMSC	Human bone marrow-derived mesenchymal stromal cells
hFMSC	Human femoral-derived mesenchymal stromal cells
HLA-DR	Human leukocyte antigen - DR isotype
hMSC	Human mesenchymal stromal cells
ISCT	International Society for Cellular Therapy
mRNA	Messenger ribonucleic acid
MSC	Mesenchymal stromal cells
oAMSC	Ovine adipose tissue-derived mesenchymal stromal cells
oBMSC	Ovine bone marrow-derived mesenchymal stromal cells
OD	Optical density
oFMSC	Ovine femoral-derived mesenchymal stromal cells
oMSC	Ovine mesenchymal stromal cells
PBMC	Peripheral blood mononuclear cell
PBS	Phosphate-buffered saline
PFA	Paraformaldehyde
Pi	Phosphate ions
PMA	Phorbol myristate acetate
RNA	Ribonucleic acid
RT-PCR	Real-time polymerase chain reaction
Runx2	Runt-related transcription factor 2
